# Supplementary material for: Identification of a sub-population of synovial mesenchymal stem cells with enhanced treatment efficacy in a rat model of osteoarthritis
Source: eLife. 2026 Jan 20;14:RP103332. doi: 10.7554/eLife.103332 (PMC12818869; doi:10.7554/eLife.103332)
Supplement: Supplementary file 1. — There was no difference in the ages between the normal and OA group (P=0.13). [file elife-103332-supp1.docx]

**Supplementary File 1.** Summary of the participants in the study. There was no difference in the ages between the normal and OA group (*p*=0.13).

|  | **Total Number (Male and Female)** | **Age Range** |
| --- | --- | --- |
| **Normal** | **18 (14M/4F)** | **19-77** |
| **OA** | **15 (5M/10F)** | **46-83** |
