# Supplementary material for: Identification of a sub-population of synovial mesenchymal stem cells with enhanced treatment efficacy in a rat model of osteoarthritis
Source: eLife. 2026 Jan 20;14:RP103332. doi: 10.7554/eLife.103332 (PMC12818869; doi:10.7554/eLife.103332)
Supplement: Supplementary file 3. [file elife-103332-supp3.docx]

**Supplementary File 3.** Summary of the self-renewal capacity (population doublings) from all clonal lines derived in the study.

| **Population Doublings** | **<10** | **10 - 15** | **15 - 18** | **18 - 20** | **>20** |
| --- | --- | --- | --- | --- | --- |
| **# Normal Clones** | **69** | **54** | **45** | **31** | **29** |
| **# OA Clones** | **94** | **53** | **39** | **36** | **37** |
