# Supplementary material for: Identification of a sub-population of synovial mesenchymal stem cells with enhanced treatment efficacy in a rat model of osteoarthritis
Source: eLife. 2026 Jan 20;14:RP103332. doi: 10.7554/eLife.103332 (PMC12818869; doi:10.7554/eLife.103332)
Supplement: Supplementary file 5. [file elife-103332-supp5.docx]

**Supplementary File 5.** Summary of the cell surface marker expression (in situ and in vitro) and differentiation potential of clones derived from OA patients.

| ***Cell Potency*** | **Cell Surface Marker Expression In-Situ** | | | | | **Chondrogenic Capacity** | **Osteogenic Capacity** | **Adipogenic Capacity** | **Cell Surface Marker Expression In-Vitro** | | | | |
| --- | --- | --- | --- | --- | --- | --- | --- | --- | --- | --- | --- | --- | --- |
|  | **CD90** | **CD44** | **CD73** | **CD105** | **CD271** |  |  |  | **CD90** | **CD44** | **CD73** | **CD105** | **CD271** |
| MPCs (13) | Positive | Positive | Positive | Negative | Negative | Positive | Positive | Positive | Positive | Positive | Positive | Positive | Negative |
|  | Positive | Positive | Positive | Negative | Negative | Positive | Positive | Positive | Positive | Positive | Positive | Positive | Negative |
|  | Positive | Positive | Positive | Negative | Negative | Positive | Positive | Positive | Positive | Positive | Positive | Positive | Negative |
|  | Positive | Positive | Positive | Negative | Negative | Positive | Positive | Positive | Positive | Positive | Positive | Positive | Negative |
|  | Positive | Positive | Positive | Negative | Negative | Positive | Positive | Positive | Positive | Positive | Positive | Positive | Negative |
|  | Positive | Positive | Positive | Negative | Negative | Positive | Positive | Positive | Positive | Positive | Positive | Positive | Negative |
|  | Negative | Positive | Positive | Negative | Negative | Positive | Positive | Positive | Positive | Positive | Positive | Positive | Negative |
|  | Negative | Positive | Positive | Negative | Negative | Positive | Positive | Positive | Positive | Positive | Positive | Positive | Negative |
|  | Negative | Positive | Positive | Negative | Negative | Positive | Positive | Positive | Positive | Positive | Positive | Positive | Negative |
|  | Negative | Positive | Positive | Negative | Negative | Positive | Positive | Positive | Positive | Positive | Positive | Positive | Negative |
|  | Negative | Positive | Positive | Negative | Negative | Positive | Positive | Positive | Positive | Positive | Positive | Positive | Negative |
|  | Negative | Positive | Negative | Negative | Negative | Positive | Positive | Positive | Positive | Positive | Positive | Positive | Negative |
|  | Negative | Positive | Positive | Positive | Positive | Positive | Positive | Positive | Positive | Positive | Positive | Positive | Positive |
| Bi- potent Progenitors (9) | Positive | Positive | Positive | Negative | Negative | Positive | Negative | Positive | Positive | Positive | Positive | Positive | Negative |
|  | Positive | Positive | Positive | Negative | Negative | Positive | Negative | Positive | Positive | Positive | Positive | Positive | Negative |
|  | Positive | Positive | Positive | Negative | Negative | Positive | Negative | Positive | Positive | Positive | Positive | Positive | Negative |
|  | Positive | Positive | Positive | Positive | Negative | Positive | Negative | Positive | Positive | Positive | Positive | Positive | Negative |
|  | Negative | Positive | Positive | Negative | Negative | Positive | Negative | Positive | Positive | Positive | Positive | Positive | Negative |
|  | Negative | Positive | Negative | Negative | Negative | Positive | Positive | Negative | Positive | Positive | Positive | Positive | Negative |
|  | Positive | Positive | Positive | Positive | Negative | Positive | Positive | Negative | Positive | Positive | Positive | Positive | Negative |
|  | Positive | Positive | Positive | Negative | Negative | Negative | Positive | Positive | Positive | Positive | Positive | Positive | Negative |
|  | Negative | Positive | Negative | Negative | Negative | Negative | Positive | Positive | Positive | Positive | Positive | Positive | Negative |
| Uni- potent Progenitors (9) | Positive | Negative | Positive | Negative | Negative | Positive | Negative | Negative | Positive | Positive | Positive | Positive | Negative |
|  | Positive | Positive | Positive | Negative | Negative | Negative | Positive | Negative | Positive | Positive | Positive | Positive | Negative |
|  | Negative | Positive | Positive | Negative | Negative | Negative | Negative | Positive | Positive | Positive | Positive | Positive | Negative |
|  | Positive | Positive | Positive | Negative | Negative | Negative | Negative | Positive | Positive | Positive | Positive | Positive | Negative |
|  | Positive | Positive | Positive | Negative | Negative | Negative | Negative | Positive | Positive | Positive | Positive | Positive | Negative |
|  | Positive | Positive | Positive | Negative | Negative | Negative | Positive | Negative | Positive | Positive | Positive | Positive | Negative |
|  | Positive | Positive | Positive | Negative | Negative | Positive | Negative | Negative | Positive | Positive | Positive | Positive | Negative |
|  | Positive | Positive | Positive | Positive | Negative | Negative | Negative | Positive | Positive | Positive | Positive | Positive | Negative |
|  | Positive | Positive | Positive | Positive | Negative | Negative | Negative | Positive | Positive | Positive | Positive | Positive | Negative |
|  | Negative | Positive | Positive | Negative | Negative | Negative | Negative | Positive | Positive | Positive | Positive | Positive | Negative |
| No differentiation capacity (2) | Negative | Positive | Positive | Negative | Negative | Negative | Negative | Negative | Positive | Positive | Positive | Positive | Negative |
|  | Positive | Positive | Positive | Negative | Negative | Negative | Negative | Negative | Positive | Positive | Positive | Positive | Negative |
