# Supplementary material for: Identification of a sub-population of synovial mesenchymal stem cells with enhanced treatment efficacy in a rat model of osteoarthritis
Source: eLife. 2026 Jan 20;14:RP103332. doi: 10.7554/eLife.103332 (PMC12818869; doi:10.7554/eLife.103332)
Supplement: Supplementary file 6. [file elife-103332-supp6.docx]

**Supplementary File 6.** Summary of marker expression and differentiation potential from CD90^+^CD44^+^CD73^+^ cells derived from normal and OA synovium.

| ***Cell Potency*** | **Patient** | **CD90^+^CD44^+^CD73^+^** | **Chondrogenic Capacity** | **Osteogenic Capacity** | **Adipogenic Capacity** | **Cell Surface Marker Expression In-Vitro** | | | | |
| --- | --- | --- | --- | --- | --- | --- | --- | --- | --- | --- |
|  |  |  |  |  |  | **CD90** | **CD44** | **CD73** | **CD105** | **CD271** |
| **Normal Synovium** | **1** | **Yes** | **Negative** | **Positive** | **Positive** | **Positive** | **Positive** | **Positive** | **Positive** | **Negative** |
|  | **1** | **No** | **Negative** | **Negative** | **Negative** | **Positive** | **Positive** | **Positive** | **Positive** | **Negative** |
|  | **2** | **Yes** | **Positive** | **Negative** | **Positive** | **Positive** | **Positive** | **Positive** | **Positive** | **Negative** |
|  | **2** | **No** | **Negative** | **Negative** | **Positive** | **Positive** | **Positive** | **Positive** | **Positive** | **Negative** |
|  | **3** | **Yes** | **Positive** | **Positive** | **Positive** | **Positive** | **Positive** | **Positive** | **Positive** | **Negative** |
|  | **3** | **No** | **Positive** | **Negative** | **Negative** | **Positive** | **Positive** | **Positive** | **Positive** | **Negative** |
|  | **4** | **Yes** | **Negative** | **Positive** | **Positive** | **Positive** | **Positive** | **Positive** | **Positive** | **Negative** |
|  | **4** | **No** | **Positive** | **Negative** | **Positive** | **Positive** | **Positive** | **Positive** | **Positive** | **Negative** |
| **OA Synovium** | **1** | **Yes** | **Negative** | **Positive** | **Positive** | **Positive** | **Positive** | **Positive** | **Positive** | **Negative** |
|  | **1** | **No** | **Negative** | **Negative** | **Positive** | **Positive** | **Positive** | **Positive** | **Positive** | **Negative** |
|  | **2** | **Yes** | **Positive** | **Negative** | **Negative** | **Positive** | **Positive** | **Positive** | **Positive** | **Negative** |
|  | **2** | **No** | **Positive** | **Positive** | **Positive** | **Positive** | **Positive** | **Positive** | **Positive** | **Negative** |
|  | **3** | **Yes** | **Negative** | **Negative** | **Positive** | **Positive** | **Positive** | **Positive** | **Positive** | **Negative** |
|  | **3** | **No** | **Positive** | **Positive** | **Positive** | **Positive** | **Positive** | **Positive** | **Positive** | **Negative** |
|  | **4** | **Yes** | **Negative** | **Positive** | **Positive** | **Positive** | **Positive** | **Positive** | **Positive** | **Negative** |
|  | **4** | **No** | **Negative** | **Negative** | **Negative** | **Positive** | **Positive** | **Positive** | **Positive** | **Negative** |
